# The protective role of vitamin D in BNT162b2 vaccine-related acute myocarditis

**DOI:** 10.3389/fimmu.2025.1501609

**Published:** 2025-02-19

**Authors:** Hing Wai Tsang, Gilbert T. Chua, Keith Tsz Suen Tung, Rosa Sze Man Wong, Sabrina Siu Ling Tsao, Joshua Sung Chih Wong, Joanna Yuet Ling Tung, Janette Siu Yin Kwok, Jason Cheuk Sing Yam, Godfrey Chi Fung Chan, Kelvin Kai Wang To, Ian Chi Kei Wong, Wing Hang Leung, Mike Yat Wah Kwan, Patrick Ip

**Affiliations:** ^1^ Department of Paediatrics and Adolescent Medicine, School of Clinical Medicine, The University of Hong Kong, Hong Kong, Hong Kong SAR, China; ^2^ Department of Special Education and Counselling (SEC), The Education University of Hong Kong, Hong Kong, Hong Kong SAR, China; ^3^ Department of Paediatrics and Adolescent Medicine, Princess Margaret Hospital, Hospital Authority, Hong Kong, Hong Kong SAR, China; ^4^ Department of Paediatrics and Adolescent Medicine, Hong Kong Children’s Hospital, Hong Kong, Hong Kong SAR, China; ^5^ Department of Pathology, Queen Mary Hospital, Hospital Authority, Hong Kong, Hong Kong SAR, China; ^6^ Department of Ophthalmology and Visual Sciences, Faculty of Medicine, The Chinese University of Hong Kong, Hong Kong, Hong Kong SAR, China; ^7^ Paediatric Haematology and Oncology Centre, Hong Kong Sanatorium and Hospital, Hong Kong, Hong Kong SAR, China; ^8^ Department of Microbiology, School of Clinical Medicine, The University of Hong Kong, Hong Kong, Hong Kong SAR, China; ^9^ Department of Pharmacology and Pharmacy, Li Ka Shing Faculty of Medicine, The University of Hong Kong, Hong Kong, Hong Kong SAR, China; ^10^ School of Pharmacy, Medical Sciences Division, Macau University of Science and Technology, Macau, Macao SAR, China; ^11^ School of Pharmacy, Aston University, Brimingham, United Kingdom

**Keywords:** vitamin D, BNT162b2 vaccine-related myocarditis, mRNA COVID-19 vaccines, natural killer cell, vitamin D deficiency, hyperinflammation, hypercytokinemia, vitamin D genetics

## Abstract

**Introduction:**

Vaccine-related myocarditis is recognized as a rare but important complication, especially after mass-scale mRNA COVID-19 vaccination. Knowledge regarding how to minimize the risk is limited. As NK cells can mediate acute myocarditis after mRNA COVID-19 vaccination and vitamin D may inhibit NK cells via cytokine modulation, we hypothesize that the myocarditis side effect is related to a hypovitaminosis D – mRNA vaccine – hypercytokinemia – NK cell axis, which is amendable to clinical intervention.

**Methods:**

Biochemical, immunophenotypic and genotyping assays were performed to examine vitamin D status and immune profiles in 60 patients who had BNT162b2 vaccine-related acute myocarditis.

**Results:**

A high incidence of hypovitaminosis D (73.3%) was observed in these individuals with vaccine-related myocarditis, particularly in those presented with chest pain or intensive care unit (ICU) admission. Moreover, vitamin D level was negatively associated with peak serum cardiac troponin T level during vaccine-related myocarditis. Genotypically, the *GC* (vitamin D binding protein) rs4588T allele which encoded the *GC2* isoform of vitamin D binding protein was a risk allele, whereas the *GC1S* isoform was protective. Mechanistically, hypovitaminosis D was associated with higher levels of cytokines pivotal for natural killer (NK) cells (particularly interleukin-1β (IL-1β), IL-12, Interferon-γ (IFN-γ), and IL-8) and higher percentage of CD69+ NK cells in blood, which in turn correlated with chest pain presentation.

**Conclusion:**

These data support the hypothesis that vitamin D plays a crucial role in mitigating mRNA vaccine-related myocarditis by modulating proinflammatory cytokine milieu and subsequent unfavorable NK cell activation, laying a groundwork for preventive and treatment strategies.

## Introduction

COVID-19 vaccines have been shown to be highly effective against SARS-CoV-2 infection in real-world settings (1). COVID-19 vaccination programs have now been implemented worldwide for several years, with more than 13.6 billion vaccine doses administered (2).

Following the mass administration of COVID-19 vaccinations worldwide, different adverse side effects were observed and reported after the first, second, or booster doses of the different COVID-19 vaccines. In particular, acute myocarditis has been recognized as a rare but important specific complication following mRNA COVID-19 vaccinations (3–5). The immune-mediated adverse events following mRNA vaccinations are generally accepted to be associated with an exaggerated inflammatory response to the molecular mimicry of the antigen present in the mRNA vaccines (6, 7). Susceptible individuals with underlying factors may present with a rapid, amplified, and prolonged inflammatory response after vaccination, with elevated levels of pro-inflammatory cytokines, such as, interleukin-18 (IL-18), IL-1β and IL-15 (8, 9). Our group was the first to show that NK cells were involved in the pathogenic mechanism of BNT162b2 vaccine-related myocarditis. We demonstrated that KIR genes and NK cell cytotoxic genes contributed to the observed expansion of CD57+ NK subsets in a BNT162b2 vaccine-related myocarditis patients cohort. Moreover, we found a prominent expansion of the identified NK cell subset in male patients or in those receiving the second dose of mRNA COVID-19 vaccine. However, an underlying explanation for these vulnerability factors and how NK cells were selectively activated have not been elucidated (10).

As vitamin D level has been linked to COVID-19 severity via its regulation of pro-inflammatory cytokines and 1,25(OH)D3 has been shown to have a direct inhibitory effect on NK cytotoxicity in a dose-dependent manner, we hypothesized that sufficient vitamin D levels play a critical protective role in minimizing the exaggerated hyperinflammatory response and the undesirable NK cell reactions after mRNA vaccines, thereby reducing the risk of vaccine-related myocarditis (11–13). At present, very few published studies have investigated the role of vitamin D in minimizing the risk and progression of vaccine-related myocarditis.

## Materials and methods

### Subject recruitment

This study was conducted in a patient cohort that aimed at identifying all suspected cases of acute myocarditis in adolescents who received the BNT2162b2 vaccine, and the findings was reported earlier in our published studies (10, 14). Individuals who received the mRNA COVID-19 vaccine consented to link their electronic health records from the Hong Kong Hospital Authority (HA), the major publicly funded healthcare provider, to their vaccination records through the COVID-19 vaccines Adverse events Response and Evaluation (CARE) program. Between July 2021 to June 2022, individuals who aged between 12-17 years with suspected post-vaccine related acute myocarditis who had received the 1^st^, 2^nd^ and 3^rd^ dose of mRNA COVID-19 vaccine within 14 days prior to admission to one of the HA hospitals were reported to the Advanced Incident Reporting System (AIRS) on admission, a system for HA to report adverse drug events to the Department of Health, HKSAR Government. The subjects were recruited at the time of hospital admission and participants’ information on age, sex and ethnicity was self-reported. The suspected cases were managed by clinicians according to the Hong Kong Paediatrics Investigation Protocol for Comirnaty-related Myocarditis/Pericarditis. At the time of admission, all patients were serially monitored for potential cardiac abnormalities by electrocardiogram (ECG), echocardiogram, and serum cardiac troponin T levels. The ECGs were interpreted by a single investigator (S.S.T.), whereas echocardiograms were performed and analyzed by the cardiologists at the admitting hospital. Cardiac magnetic resonance imaging (cMRI) was conducted to confirm cases upon admission to hospital or at the Hong Kong Children’s Hospital within two weeks of symptoms onset. Radiologists at the MRI unit interpreted the images according to Lake Louise Myocarditis Criteria 2018 (15). All cases of BNT162b2 vaccine-related myocarditis reported in the study were later confirmed by the study team following the guidelines listed in the Brighton Collaboration Case Definition of Myocarditis and Pericarditis (16). Moreover, 9 convalescent patient samples were collected during their follow-up clinics after recovery from myocarditis. (**Supplementary Table 1**). Patients were excluded if they subsequently tested positive for SARS-CoV-2, influenza A/B/C, parainfluenza virus 1/2/3/4, adenovirus, human metapneumovirus, respiratory syncytial virus, or enterovirus. Those with a past history of COVID-19 confirmed by SARS-CoV-2 receptor binding domain (RBD) antibody and nucleocapsid protein (NP) antibody testing were also excluded from this study.

A representative local cohort, composed of 378 Hong Kong healthy infants in a previously published study, were included to estimate the population frequencies of vitamin D-binding protein genetic polymorphisms (17). Infants aged between 2 – 12 months were recruited in 5 main districts Maternal and Child Health Centers in Hong Kong using stratified sampling from July 2019 to May 2021 and the obtained allelic and genotypic frequency results aligned with other published studies among Han Chinese population (18–21). Infants with major congenital malformations, conditions including low birth weight and premature birth, with known genetic diseases, chronic medical problems were excluded.

### Isolation of serum and peripheral blood mononuclear cells

Whole blood samples were collected from patients during hospitalization for vaccine-related myocarditis and during the recovery period. Serum samples were recovered by centrifugation, aliquoted, and stored at -80°C until analysis. PBMCs were isolated from heparinized blood by the gradient density centrifugation method and cryopreserved. The samples were recovered in batches for immunophenotyping.

### Measurement and definition of vitamin D deficiency and insufficiency

Serum 25(OH)D levels were determined by liquid chromatography-tandem mass spectrometry (LC-MS/MS). An AB Sciex Triple Quad QTRAP 5500+ LC-MS/MS system (AB Sciex Pte. Ltd., Framingham, MA) was used to simultaneously detect levels of cholecalciferol (25(OH)D3), ergocalciferol (25(OH)D2), and 3-epi-25 hydroxyvitamin D (3-epi-25(OH)D3). Total serum 25(OH)D level, defined as the sum of 25(OH)D3 and 25(OH)D2 adjusted by 3-epi-25(OH)D3, was determined by the standard curve method using accredited standard solutions (MilliporeSigma, St. Louis, MO). Data obtained through the LC/MS-MS method in this study have been certified by the external quality assurance program conducted by the vitamin D External Quality Assessment Scheme (DEQAS, Endocrine Laboratory, Charing Cross Hospital, London, UK) (22). Vitamin D sufficiency was defined as serum 25(OH)D > 50 nmol/L; vitamin D insufficiency was defined as 50 nmol/L ≥ serum 25(OH)D ≥ 25 nmol/L; and vitamin D deficiency was defined as serum 25(OH)D <25 nmol/L (23).

### Cardiac troponin T measurement

Serum cardiac troponin T concentration was measured using the Human Cardiac Troponin T ELISA Kit (Abcam, Cambridge, United Kingdom) using the standard curve method according to the manufacturer’s protocol.

### Cytokine measurements

Levels of serum cytokines were measured using pre-designed LEGENDplex™ cytokine panels (Biolegend, San Diego, CA). This is a bead-based immunoassay targeting pro-inflammatory cytokines (IL-1β, IFN-α2, IFN-γ, TNF-α, MCP-1, IL-6, IL-8, IL-10, IL-12p70, IL-17A, IL-18, IL-23, and IL-33) and selected T helper cytokines (IL-2, IL-4, IL-5, IL-9, IL-13, and IL-22). Fluorescence intensity was captured by a BD LSR-II flow cytometer (BD Bioscience, San Jose, CA) and analyte concentration was estimated by the standard curved method.

### Immunophenotyping of PBMCs

The recovered PBMCs were stained with viability dye (VioGreen™, Miltenyi Biotechnology, Germany) before fixation for further analysis. Cells were cold-fixed, permeabilized, and labeled with an antibody cocktail containing Pacific Blue anti-human CD3 (Clone no.: HIT3a, Biolegend), APC anti-human CD56 (Clone no.:5.1H11, Biolegend), and APC/Cyanine 7 anti-human CD14 (Clone no.:M5E2, Biolegend) for the identification of NK cells and monocytes, and exclusion of T cells; and FITC anti-human CD69 (Clone no.: FN50, Biolegend) and PE anti-human HLA-DR (Clone no.: LN3, Biolegend) for the corresponding activation analyses. The stained cells were analyzed by a BD LSR-II flow cytometer (BD Biosciences) and 100,000 representative events were captured. Flow gating was applied to select immune cells as illustrated in **Supplementary Figure 1**.

### DNA extraction and genotyping of vitamin D genetic variants

Leukocytic DNA was extracted from heparinized blood samples by QIAamp^®^ DNA Mini Kit (QIAGEN, Venlo, Netherlands). Four common genetic variants, rs7041, rs4588, rs2282679, and rs2228570, which are significantly associated with circulating vitamin D levels, were genotyped to study their association with the risk of BNT162b2 vaccine-related myocarditis (24–27). Genotyping was performed by Taqman-based SNP assay (ThermoFisher Scientific, Waltham, MA) according to the manufacturer’s protocol.

Restriction Fragment Length Polymorphism (RFLP) was performed to identify the rs7041-rs4588 haplotype or different GC isoforms in patients and controls. Primer pairs (forward primer: 5′-CTGGACTTCCAATTCAGCAG-3′; reverse primer: 5′-AATGGCATCTCAATAACAGG-3′) were designed to generate an amplicon from the subject’s genomic DNA including rs7041 and rs4588 loci by PCR, followed by double restriction digestion by 1U StyI and HaeIII (New England Biolabs Inc., Ipswich, MA, USA). The GC isoform was detected by 2% agarose gel electrophoresis (**Supplementary Figure 2**).

### Quantification and statistical analyses

Statistical analyses were performed using SPSS for Windows (version 27.0, SPSS Inc., Chicago, IL) and Prism for Windows (version 8.0.1, GraphPad Software, San Diego, CA). Comparative analysis and correlation analysis with p<0.05 were considered statistically significant. Immunophenotyping data analysis was performed using FlowJo (version 10.1, BD Biosciences). LEGENDplex™ data analysis software suite (version 2023-02-15, Biolegend) was used to estimate cytokine concentrations. Cohen’s d was calculated to quantify differences in cTnT levels between risk and non-risk haplotype carriers, as well as cytokine levels between vitamin D deficiency/insufficiency and sufficiency groups (28). Odds ratio was used to assess the strength of the association to the risk of BNT162b2 vaccine-related myocarditis. Spearman’s correlation analysis was performed to analyze the relationship between 25(OH)D level, risk of symptoms onset, and cTnT levels in patients with BNT162b2 vaccine-related myocarditis. In addition, the relationship between the selected NK cell and monocyte subsets to the presented symptoms and vitamin D levels was analyzed by Spearman’s test and expressed as Spearman’s Rank correlation coefficient (ρ) and p-value in the association analysis.

### Ethics approval

The study was approved by the Institutional Review Board of the University of Hong Kong/Hospital Authority Hong Kong West Cluster (Reference: UW21-149 and UW21-138) and the Department of Health Ethics Committee (LM21/2021). Written consent was obtained from the parents or legal guardians of the subjects.

## Results

### Subject recruitment and clinical characteristics

Between 1^st^ July 2021 to 30^th^ June 2022, 50 males and 10 female patients who were diagnosed with BNT162b2 vaccine-related myocarditis were recruited from public hospitals in Hong Kong SAR. Demographics and presenting clinical characteristics of subjects during the acute myocarditis period are shown in **Table 1**. All the recruited subjects were Han Chinese. Fifty (83.3%) patients were male with a median age of 15 years and 10 (16.7%) patients were female with a median age of 13 years. Patients showed symptoms on a median of 3 days after receiving mRNA COVID-19 vaccination. The majority (70%) of patients showed symptoms after receiving the second dose of the vaccine. Most patients presented with chest pain (88.3%), followed by fever and palpitation (~30%), shortness of breath (SOB) (23.3%), and dizziness and headache (~20%). Eighteen patients (30.0%) needed to be transferred to the intensive care unit (ICU) at the time of hospitalization. Cardiac monitoring showed 48 (80.0%) patients had abnormal ECG, 42 (70.0%) had abnormal cMRI, and 22 (36.7%) had abnormal echocardiogram and all patients also had elevated serum cardiac troponin T (cTnT) level. All patients presented mild symptoms that either required no treatment or were alleviated through the use of non-steroidal anti-inflammatory drugs. Spontaneous recovery occurred without the necessity for systemic steroids, intravenous immunoglobulins, intubation, inotropic support, or ventricular assist devices.

**Table 1:** Demographics and clinical characteristics of Comirnaty-related acute myocarditis/percarditis patients.

	Vaccine-related myocarditis patients (N=60)
Median age in years	15
Age range	12-17
Gender (%)/Median Age	
Male	83.3 (50/60)/15
Female	16.7 (10/60)/13
Ethnicity (%)	
Han Chinese	100 (60/60)
Others	0
Median days of hospital admission from the last dose/range	3 (1-14)
Dose(s) of BNT162b2 received before symptoms onset(%)	
1^st^ dose	15.0 (9/60)
2^nd^ dose	70.0 (42/60)
3^rd^ dose	15.0 (9/60)
Signs/Symptoms (%)	
Chest pain	88.3 (53/60)
Fever	38.8 (23/60)
Palpitation	35.0 (21/60)
Shortness of Breath (SOB)	23.3 (14/60)
Dizziness	20.0 (12/60)
Headache	18.3 (11/60)
Vomiting	6.7 (4/60)
Back pain	1.7 (1/60)
Admission to Intensive Care Unit (%)	30.0 (18/60)
Abnormal cardiac checkup (%)	
Average serum Cardiac Troponin T concentration [ng/mL] (Mean ± SD)	0.62 ± 0.40^***^
Electrocardiogram	80.0 (48/60)
Cardiac magnetic resonance imaging (cMRI)	70.0 (42/60)
Echocardiogram	36.7 (22/60)
Sample collection period	July 2021 - June 2022

***p<0.001

### Higher prevalence of hypovitaminosis D in vaccine-related myocarditis patients correlates with the outcome of the cardiac complication

**Figure 1:**
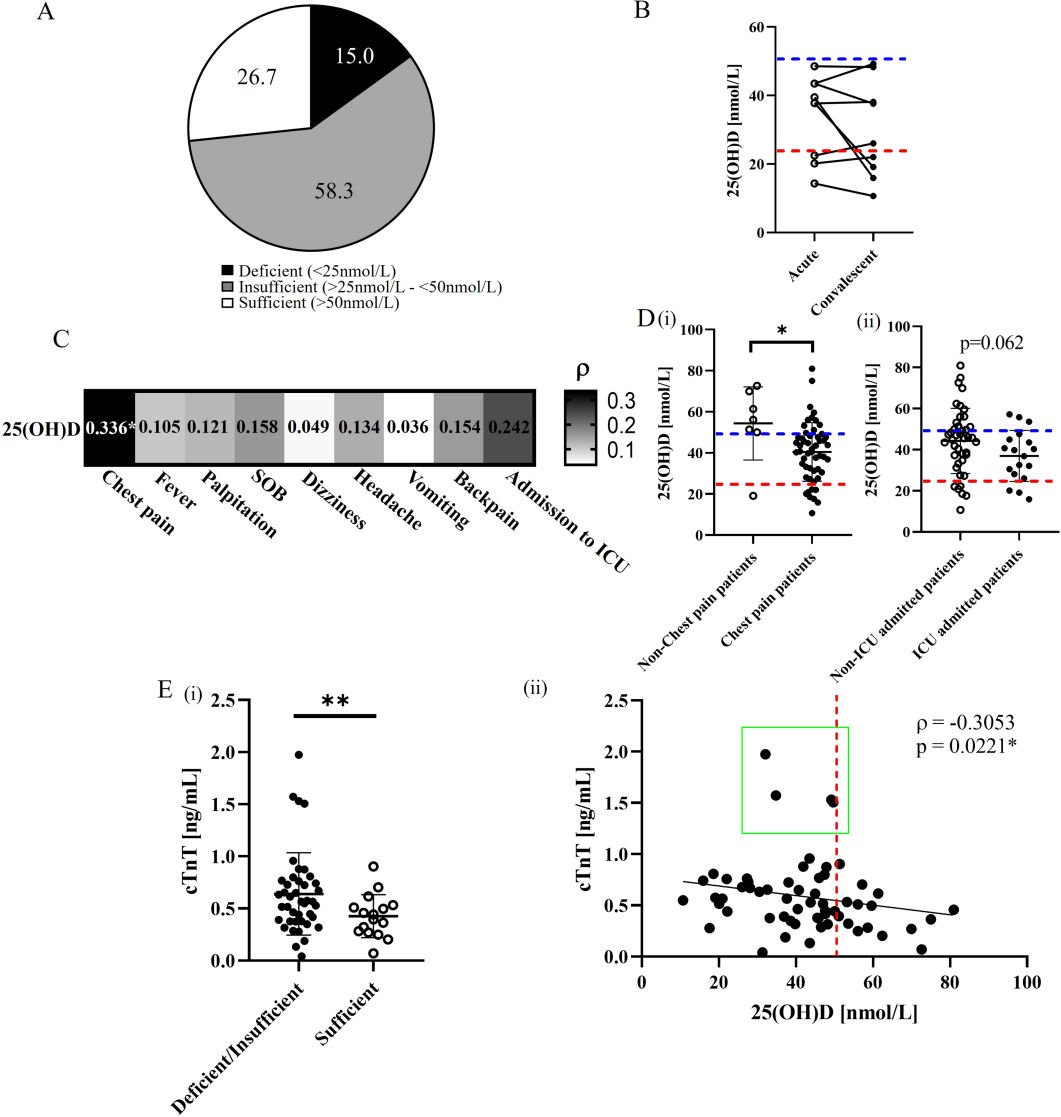
Assessment of vitamin D status and serum levels related to cardiac complications in BNT162b2 vaccine-related myocarditis. **(A)** Prevalence (%) of vitamin D deficiency, insufficiency, and sufficiency 60 BNT162b2 vaccine-related myocarditis patients. **(B)** Comparative analysis of circulating 25(OH)D levels between 9 acute and convalescent paired patient samples. Blue and red dotted lines represent the cutoffs for vitamin D insufficiency and deficiency, respectively. *p<0.05. **(C)** Spearman’s correlation analysis between 25(OH)D levels and presented symptoms in vaccinated myocarditis patients. *p<0.05. **(D)** Comparative analysis of 25(OH)D levels between **(i)** patients presenting with or without chest pain and **(ii)** those admitted to the ICU or not. *p<0.05. **(E)**(i) Comparative analysis of serum cTnT levels between vitamin D deficient/insufficient and sufficient patients. **p<0.01. **(ii)** Spearman’s analysis between cTnT levels and 25(OH)D levels among patients. The red dotted line represents the cutoff between vitamin D deficiency/insufficiency and sufficiency (50 nmol/L). The green box highlights patients with relatively higher cTnT levels. *p<0.05.

A high prevalence of vitamin D deficiency or insufficiency (73.3%) was observed in the patients with BNT162b2 vaccine-related myocarditis (**Figure 1A**). In 9 patients with hypovitaminosis D and paired convalescent samples available (collected at a median of 36 days after myocarditis onset) (**Supplementary Table 1**), the 25(OH)D level remained low during recovery period, similar to that at the acute phase. (**Figure 1B**). Further analysis revealed the serum 25(OH)D level correlated with the presenting symptoms. Specifically, vitamin D level was found to be significantly associated with the risk of chest pain (ρ=0.336, p=0.015), with those presenting without chest pain having significantly higher average 25(OH)D levels (p=0.0215). A similar trend was observed for ICU admission (ρ=0.242, p=0.084) (**Figure 1C**), with non-ICU admitted patients having numerically higher 25(OH)D levels (**Figure 1D ii**), although this did not reach statistical significance (p=0.062). There was a negative association between 25(OH)D level and cTnT levels (ρ=-0.3053, p=0.0221) (**Figure 1Eii**), with significantly lower cTnT levels observed in patients with sufficient vitamin D levels compared to patients with insufficient/deficient vitamin D levels (p=0.0099) (**Figure 1Ei**). Moreover, all four patients with the highest cTnT levels had insufficient/deficient vitamin D levels (<50 nmol/L) (**Figure 1Eii**, green box). Overall, our findings support the hypothesis that sufficient vitamin D levels may reduce the risk and severity of BNT162b2 vaccine-related myocarditis, in line with the known function of vitamin D in maintaining normal immune function and modulating hyperinflammatory responses (29–32).

### Vitamin D modulates specifically NK cell cytokine profiles in BNT162b2 vaccine-related myocarditis patients

To test our hypothesis that the protective effect of vitamin D was mediated mechanistically via modulation of cytokines pivotal for NK cell activation, we further measured serum cytokine levels and correlated with vitamin D status. (**Table 2**). Compared to the vitamin D sufficient group, general pro-inflammatory cytokines such as IL-1β, IL-6, IL-8, IL-10, IL-12 were found to be significantly elevated in vitamin D deficient/sufficient groups (all Cohen’s d >0.22). Particularly, the four most significantly elevated cytokines in the deficient/insufficient group were IL-1β, IL-8, IL-12, and IFN-γ (all Cohen’s d>0.25), all of which have been shown in our prior study to be specifically associated with NK-cell mediated vaccine-induced myocarditis (10). In contrast, the pleiotropic cytokine IL-4, which is a potent direct inhibitor of NK cells, showed higher levels in the sufficient group than in deficient/insufficient group (Cohen’s d=0.3847) (33, 34). Notably, cytokines that are crucial for monocyte-mediated tissue inflammation such as monocyte chemoattractant protein-1 (MCP-1) and IL-22 did not correlate with vitamin D status (35). Taken together, these results suggest that sufficient vitamin D levels could regulate vaccine-associated exaggerated inflammation via a specific inhibitory cytokine profile on NK cells but not on monocytes.

**Table 2:** Selected inflammatory cytokine levels among vitamin D deficient/insufficient or vitamin D sufficient BNT162b2 vaccine-related myocarditis patients.

Cytokines		Mean ± SD	Cohen’s D	Cytokines		Mean ± SD	Cohen’s D	Cytokines		Mean ± SD	Cohen’s D
IL-1β	Def/Insuff	109.83 ± 141.00	**0.3286**	IL-10	Def/Insuff	21.02 ± 47.39	0.2200	IL-2	Def/Insuff	12.29 ± 17.41	0.1678
	Suff	72.05 ± 80.99			Suff	13.38 ± 13.01			Suff	15.43 ± 19.93	
IFN-α2	Def/Insuff	19.85 ± 24.41	0.0250	IL-12	Def/Insuff	21.01 ± 27.24	**0.3453**	IL-4	Def/Insuff	96.02 ± 194.94	**0.3847**
	Suff	19.25 ± 23.85			Suff	13.94 ± 9.84			Suff	197.41 ± 317.71	
IFN-γ	Def/Insuff	42.13 ± 51.70	**0.3444**	IL-17	Def/Insuff	51.35 ± 96.65	0.1319	IL-5	Def/Insuff	25.80 ± 45.74	0.1428
	Suff	28.09 ± 25.54			Suff	40.44 ± 65.99			Suff	20.73 ± 20.71	
TNF-α	Def/Insuff	142.77 ± 425.83	0.2308	IL-18	Def/Insuff	510.49 ± 293.87	0.0682	IL-9	Def/Insuff	46.55 ± 101.40	0.0532
	Suff	71.30 ± 102.48			Suff	528.90 ± 243.70			Suff	51.14 ± 67.89	
MCP-1	Def/Insuff	206.79 ± 113.26	0.1934	IL-23	Def/Insuff	43.86 ± 75.19	0.1190	IL-13	Def/Insuff	19.45 ± 25.70	0.1864
	Suff	187.58 ± 83.20			Suff	36.55 ± 43.48			Suff	24.40 ± 27.34	
IL-6	Def/Insuff	27.07 ± 37.11	0.2238	IL-33	Def/Insuff	490.64 ± 589.20	0.1009	IL-22	Def/Insuff	29.00 ± 45.54	0.1272
	Suff	20.53 ± 18.26			Suff	555.97 ± 701.35			Suff	35.72 ± 59.27	
IL-8	Def/Insuff	220.35 ± 399.83	**0.2861**								
	Suff	135.39 ± 128.71									

Bold: cytokines specifically associated with NK cells.

### CD69+ NK cell expansion associated with vitamin D level in patients with BNT162b2 vaccine-related myocarditis

**Figure 2:**
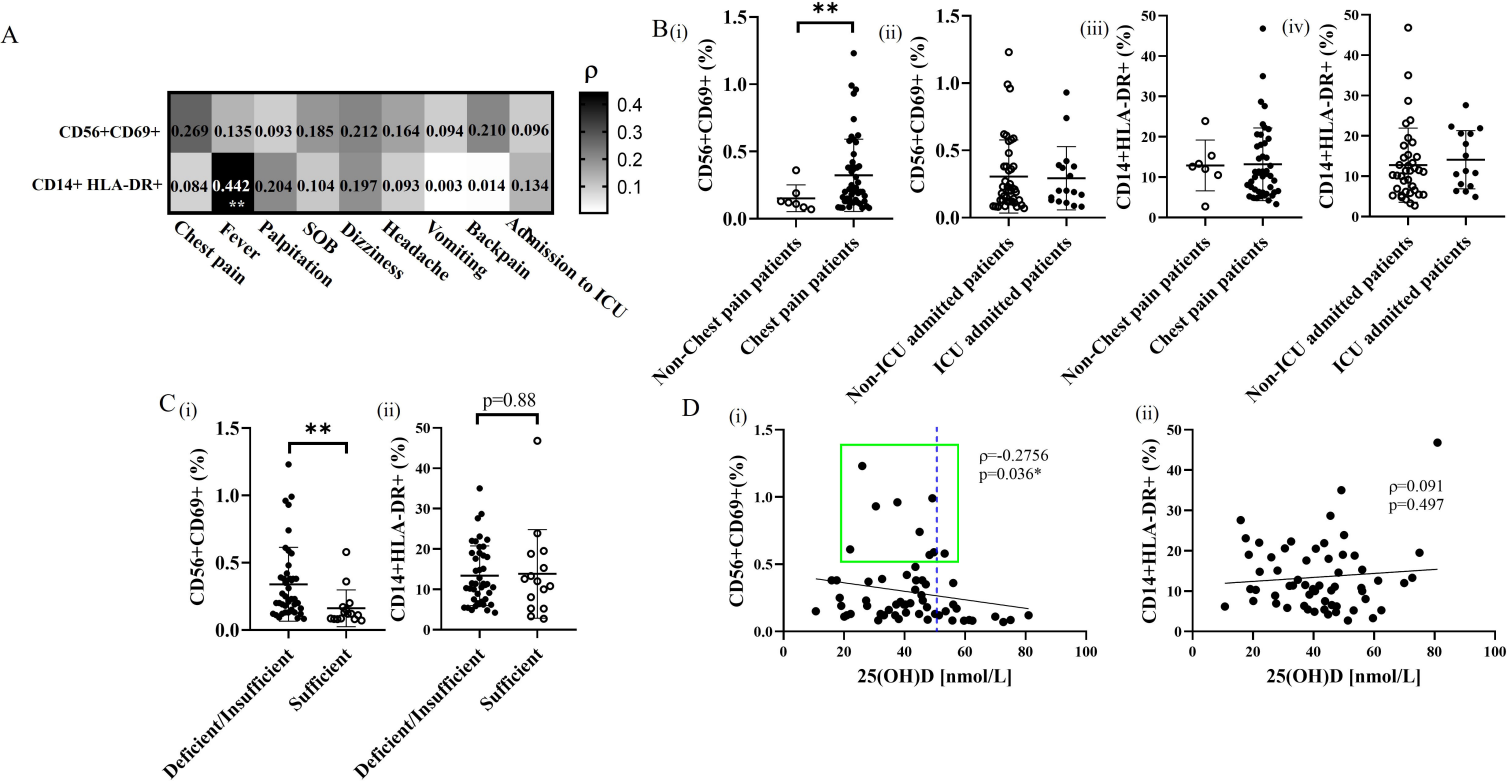
Correlation and qualitative comparison of activation subsets of NK cells and monocytes in BNT162b2 vaccine-related myocarditis patients with different vitamin D status. **(A)** Association of measured frequencies of activation subsets of NK cells and monocytes with the occurrence of the initial presented symptoms in BNT162b2 vaccine-related myocarditis patients. **p<0.01. **(B)** Comparative analysis of CD69+ NK cell and HLA-DR+ monocyte subsets between **(i,iii)** patients presenting with or without chest pain and (ii,iv) those admitted to the ICU or not. **p<0.01. **(C)** Qualitative comparison of activation frequency of **(i)** NK cell and **(ii)** monocyte subsets in vitamin D deficient/insufficient patients, and vitamin D sufficient patients. Data are presented as mean ± SD and analyzed using a two-sided Student’s t-test. **p<0.01. **(D)** Spearman’s correlation analysis of activated subset frequencies of **(i)** NK cells and **(ii)** monocytes plotted against 25(OH)D levels in BNT162b2 vaccine-related myocarditis patients. The best-fit trendline is shown. *p<0.05.

As we observed significant differences in myocarditis-associated NK cell-specific cytokine profile in patients with different vitamin D status, we subsequently measured the early activation antigen CD69 expression on NK cells from patients with BNT162b2 vaccine-related myocarditis to confirm its correlation with clinical features and vitamin D status, in contrast to the non-specific activated HLA-DR+ monocyte subset (**Figure 2**). While the frequency of CD69+ NK cells in blood correlated with myocarditis-specific symptoms of chest pain (ρ=0.269; **Figure 2A**, upper panel), the frequency of HLA-DR+ monocytes correlated with non-specific febrile reaction instead (ρ=0.442; p=0.001, lower panel). There was significantly higher frequency of CD69+ NK cell subsets in patients with chest pain compared to non-chest pain subjects (p=0.0041) (**Figure 2Bi**), with subset frequency the highest in deficient/insufficient vitamin D groups (p<0.0001) compared to the control group (**Figure 2Ci**). The frequency of CD69+ NK cells was inversely correlated with serum 25(OH)D level generally (ρ=-0.2756; p=0.036, **Figure 2Di**), and was low in subjects with adequate 25(OH)D level >50 nmol/L uniformly. In contrast, there was no significant difference in the frequency of monocyte HLA-DR+ subsets in patient groups with or without chest pain, ICU admission (**Figures 2Biii**, **Biv**) or with different vitamin D status (**Figure 2Cii**) and 25(OH)D levels (**Figure 2Dii**). Overall, these results confirm the central role of NK cells in mediating the specific symptoms of BNT162b2 vaccine-related myocarditis and highlight the immunoregulatory role of vitamin D in modulating the specific CD69+ NK cell subset but not the non-specific monocyte-mediated febrile reaction.

### Correlating common genetic determinants of circulating 25(OH)D level to the risk of BNT162b2 vaccine-related myocarditis

Vitamin D metabolites are transported by vitamin D binding protein (DBP) which is encoded by the *GC* gene, whereas vitamin D receptor is encoded by the *VDR* gene. Previous studies have identified that genetic polymorphism of *GC* and *VDR* were associated with circulating 25(OH)D levels and responsiveness of vitamin D receptor (36). We next associated the selected genetic loci in patients with BNT162b2 vaccine-related myocarditis to a Han Chinese representative cohort for prediction of vitamin D-associated genetic risk of vaccine side effects (**Table 3**). In the 52 genotyped patients, increased risk of BNT162b2 vaccine-related myocarditis was observed for rs4588T carriers (OR=1.6340; 95%Cl=1.0559 – 2.5288; p=0.0275) and potentially for rs7041A (OR=1.4068; 95%Cl=0.8585-2.2790); [**Table 2(i)**]. Its genetic effects were found to be the most prominent in the homozygous risk alleles carriers (OR>1.33) of the 3 identified genetic loci whereas the rs4588G alleles (OR=0.6120; 95%Cl=0.3954 – 0.9471; p=0.0275) [**Table 3(i)**] and the homozygous rs4588GG (OR=0.5560; 95%Cl=0.3092 – 0.9996; p=0.0498) carriers was observed as protective. [**Table 3(ii)**] Considering the rs4588T haplotypes determines the GC2 isoform (rs7041A-rs4588T), BNT162b2 vaccine-related myocarditis risk was significantly associated with GC2 (OR=1.6340; 95%Cl=1.0559 – 2.5288; p=0.0275), whereas the GC1S (encoded by the non-risk rs7041C-rs4588G) appeared to be protective (OR=0.6847; 95%Cl=0.4216 – 1.1208; p=0.1329) [**Table 2(i)**]. The combined genetic effects were estimated by comparing patients carrying the identified risk haplotypes (GC2-rs2282679G-rs2228570C) to those with non-risk haplotypes (GC1S-rs2282679T-rs2228570T) [**Table 3(iii)**]. Risk haplotype carriers exhibited lower average 25(OH)D levels (Cohen’s d=0.837) and higher average cTnT levels (Cohen’s d=0.859), whereas the opposite was observed in non-risk haplotype carriers, suggesting that vitamin D genetic polymorphisms may predispose individuals to BNT162b2 vaccine-related myocarditis.

**Table 3:** The associated vitamin D genetic risk in selected variants’ (i) alleles, (ii) genotypes and, (iii) the cumulative genetic effects in BNT162b2 vaccine-related myocarditis.

(i)					
Genetic variants	Allele	Allele Frequency (n)		Odds Ratio (95%Cl)	p-value
		Patients (n=104)	Controls (n=756)		
GC rs7041	A	0.769 (80)	0.696 (526)	1.4068 (0.8685 – 2.2790)	0.1655
	C	0.231 (24)	0.294 (222)	0.7108 (0.4388 – 1.1515)	
GC rs4588	G	0.654 (68)	0.755 (571)	0.6120 (0.3954 – 0.9471)	0.0275*
	T	0.346 (36)	0.245 (185)	1.6340 (1.0559 – 2.5288)	
GC rs2282679	T	0.692 (72)	0.728 (550)	0.8427 (0.5394 – 1.3166)	0.4522
	G	0.308 (32)	0.272 (206)	1.1866 (0.7596 – 1.8538)	
VDR rs2228570	C	0.750 (78)	0.679 (513)	1.4211 (0.8887 -2.2722)	0.1423
	T	0.250 (26)	0.321 (243)	0.7037 (0.4401 – 1.1252)	
GC isoform	GC1F	0.424 (45)	0.463 (350)	0.8847 (0.5852 – 1.3377)	0.5615
	GC1S	0.221 (23)	0.292 (221)	0.6847 (0.4216 – 1.1208)	0.1329
	GC2	0.346 (36)	0.244 (185)	1.6340 (1.0559 – 2.5288)	0.0275*
* p<0.05					

(ii)					
Genetic variants	Genotype	Frequency (n)		Odds Ratio (95% Cl)	p-value
		Patients (n=52)	Controls (n=378)		
GC rs7041	AA	0.558 (29)	0.481 (182)	1.3301 (0.7421 - 2.3843)	0.338
	CA	0.423 (22)	0.429 (162)	0.9597 (0.5336 - 1.7260)	0.897
	CC	0.019 (1)	0.09 (30)	0.2248 (0.0300 - 1.6847)	0.1464
GC rs4588	GG	0.423 (22)	0.569 (215)	0.5560 (0.3092 - 09996)	0.0498*
	GT	0.462 (24)	0.373 (141)	1.4407 (0.8037 - 2.5828)	0.2202
	TT	0.115 (6)	0.058 (22)	2.1107 (0.8134 - 5.4768)	0.1247
GC rs2282679	TT	0.481 (25)	0.524 (198)	0.8418 (0.4712 - 1.5038)	0.5606
	TG	0.423 (22)	0.407 (154)	1.0667 (0.5930 - 1.9188)	0.8294
	GG	0.096 (5)	0.069 (26)	1.4403 (0.5275 - 3.9321)	0.4765
VDR rs2228570	CC	0.580 (30)	0.492 (186)	1.4076 (0.7835 - 2.5291)	0.2528
	CT	0.350 (18)	0.373 (141)	0.8899 (0.4844 - 1.6347)	0.7069
	TT	0.070 (4)	0.135 (51)	0.5343 (0.1848 - 1.5451)	0.2473
GC isoform	GC1F/1F	0.173 (9)	0.228 (86)	0.7107 (0.3331 - 1.5160)	0.3769
	GC1F/1S	0.250 (13)	0.262 (99)	0.9394 (0.4815 - 1.8326)	0.8545
	GC1F/2	0.288 (15)	0.209 (79)	1.5344 (0.8017 - 2.9367)	0.1961
	GC1S/S	0.019 (1)	0.079 (30)	0.2275 (0.0304 - 1.7043)	0.1495
	GC1S/2	0.154 (8)	0.164 (62)	0.9267 (0.4160 - 2.0645)	0.8522
	GC2/2	0.116 (6)	0.058 (22)	2.1107 (0.8134 - 5.4768)	0.1247
*p<0.05					

(iii)				
Haplotype	25(OH)D [nmol/L] (Mean ± SD)	Cohen’s d	cTnT [ng/mL] (Mean ± SD)	Cohen’s d
Risk (GC2-rs2282679G-rs2228570C) (N=24)	41.59 ± 13.00	0.837	0.66 ± 0.41	0.859
Non-risk (GC1S-rs2282679T-rs2228570T) (N=5)	54.68 ± 17.91		0.37 ± 0.26	

## Discussion

COVID-19 mRNA vaccine-related myocarditis is a rare and potentially fatal side effect, of which the biological mechanisms and vulnerable populations have not been fully elucidated. To the best of our knowledge, this is the first study examining the role of vitamin D in protection against mRNA COVID-19 vaccine-related acute myocarditis. Specifically, we found high prevalence of vitamin D deficiency or insufficiency (>70%) in vaccine-related myocarditis patients, particularly those presented with chest pain and need for ICU admission, and the serum vitamin D level was found to be negatively correlated with cardiac troponin T level. Herein, we further explored the underlying immune mechanisms and observed lower levels of proinflammatory cytokines, including those pivotal for NK cells, in sufficient vitamin D patients when compared to the vitamin D insufficiency/deficiency groups. The abundance of CD69+ NK subsets, which correlated with the chest pain risk, was also negatively correlated with serum vitamin D level. Vitamin D binding protein rs4588T and the encoded GC2 isoform related to vitamin D transportation were identified as genetic risk factors for vaccine-related myocarditis, whereas rs4588GG and GC1S appear to be protective. Collectively, these data strongly support the hypothesis that the fundamental immunomodulatory role of vitamin D in mRNA vaccine-related myocarditis is via suppression of pro-NK cell cytokine milieu.

Numerous studies have highlighted the critical role of Vitamin D in immune regulation. Importantly, individuals with Vitamin D deficiency are more prone to inflammatory conditions, which can lead to unregulated inflammation and extensive cellular damage (31, 37, 38). In this study, consistent Vitamin D levels seen in patients during both the disease and recovery phases suggest that Vitamin D insufficiency was pre-existing, rather than being a temporary result of COVID-19 vaccination or associated cardiac events. Our earlier studies noted an elevation of a wide variety of proinflammatory cytokines in patients with myocarditis following mRNA vaccination (10). This hypercytokinemia environment in vaccine-related myocarditis may stem from the diminished anti-inflammatory effects of Vitamin D, leading to an increased inflammatory response seen in patients with vaccine-related myocarditis. Such pre-existing susceptibility risk may be primarily driven by innate immunity, as myocarditis related to the mRNA COVID-19 vaccine typically develops rapidly within 3 to 4 days post-vaccination (14, 39). With insufficient Vitamin D levels, innate immune cells expressing VDR may lose the immunomodulatory effects of Vitamin D, such as acting through the NF-κB signaling pathway, a well-known inflammation mediator typically suppressed by VDR (40). The reduced Vitamin D-mediated NF-κB suppression may lead to the stronger expression of pro-inflammatory cytokines in various innate immune cells, such as IL-1β, IL-6, IL-8, TNF-α in macrophages, IL-12 in dendritic cells, and IFN-γ in NK cells, as seen in the Vitamin D insufficient/deficient patients in this study. Overall, these data support the hypothesis that Vitamin D insufficiency is a significant risk factor for general hypercytokinemia after mRNA vaccination. However, whether certain innate immunity subsets were responsible specifically for the myocarditis side effect remained uncertain.

Recently, we were the first group to identify NK cells as the primary mediator of BNT162b2 vaccine-related myocarditis. In patients with myocarditis, an unique cytokine signature was observed, consisting high levels of IL-1β, IL-8, IL-12, and IFN-γ. In this study, we found that the four highest ranking cytokines that were most associated with hypovitaminosis D were exactly these 4 cytokines, strongly supporting the hypothesis that vitamin D plays role in this myocarditis-specific cytokine profile pivotal for early activation of CD69+ NK cells. The non-specific febrile reaction and expansion of HLA-DR+ monocytes after mRNA vaccines, however, was distinct and did not correlate with vitamin D levels or myocarditis-associated chest pain. Notably, vitamin insufficiency did not result in a higher level of IL-22 or MCP-1, which are crucial for migration and infiltration of monocytes and macrophages in heart tissue. In contrast, myocarditis patients with adequate vitamin D had high serum levels of IL-4, which is known to be a potent inhibitor of NK cells directly in a concentration-dependent manner and may suppress the binding and cytotoxicity of NK cells in the vascular endothelium specifically, leading to lower cTnT levels and fewer patients with chest pain (33, 34, 41). Notably, CD69+ NK cells are not only an early activation subset, but also a tissue-resident NK cell subset (42). The observed prevalence of the CD69+ NK cell subset in the blood of BNT162b2 vaccine-related myocarditis patients with vitamin insufficiency might in part be related to the increase in myocardium-resident NK cells during acute myocarditis, which may in turn lead to increased circulation of CD69+ NK cells.

Our study provides evidence of genetic predisposition contributing to hypovitaminosis D, which escalates the risk and severity of vaccine-related myocarditis. We identified the GC rs4588T allele and GC2 isoform as risk factors, while the GC rs4588GG genotype and GC1S isoform appeared to be protective. Notably, the GC2 haplotype, encoded by rs7041A-rs4588T, is negatively associated with serum vitamin D levels consistently in the Han Chinese population, thus increasing susceptibility to vitamin D deficiency or insufficiency (18, 19). A potential correlation was found between lower circulatory concentrations of DBP and lower affinity to its metabolites, which might explain the reduced circulatory vitamin D in GC2 carriers compared to those carrying GC1F or GC1S haplotypes (43). Serum DBP is an important reservoir for vitamin D metabolites (44, 45). Furthermore, our cumulative genetic analysis supported the idea that the bioavailability of vitamin D could influence the severity of this adverse side effect, particularly with respect to the serum cardiac damage marker cTnT. Therefore, the identified negative vitamin D genetics may enhance the risk of hypovitaminosis D, thereby increasing the likelihood of vaccine-related myocarditis. Besides genetic factors, environmental aspects such as oral supplementation practices, sunlight exposure, diet, seasons, and non-modifiable factors like sex, age, and racial differences can also affect serum vitamin D levels in patients. This leads to variability in the risk and severity of vaccine-related myocarditis, necessitating further investigation.

We speculate that an appropriate level of sunlight exposure or taking vitamin D supplements before vaccination would provide substantial benefits to minimize the side effects of the mRNA COVID-19 vaccine, as vitamin D deficiency or insufficiency can be modified in a simple and safe approach. However, given the limited number of patients, the cross-sectional study design, and the individual variability in vitamin D levels affected by various factors (46–50), a larger patient cohort is necessary for further investigation into the likelihood of modifiable factors and health conditions in reducing the risk and severity of vaccine-related myocarditis. A longitudinal follow-up study could also be conducted to associate initial and subsequent vitamin D levels with long-term cardiac outcomes in patients, highlighting the significance of vitamin D in managing this adverse side effect. Previous research has suggested vitamin D as an adjunctive therapeutic agent for COVID-19 patients, due to its ability to decrease pro-inflammatory cytokine production (51). This same anti-inflammatory strategy could also be applied in treating patients presenting this side effect. This study paves the way for future clinical trials to explore the potential use of vitamin D in treating vaccine-related myocarditis patients.

The findings of this study need to be interpreted with the following caveats. First, the number of patients in this study was relatively small, as BNT162b2 vaccine-related myocarditis is uncommon. However, our extensive network and efficient reporting system allowed us to recruit sufficient cases within the study period. This is by far one of the largest adolescent cohorts of mRNA COVID-19 vaccine-related myocarditis, and also provides a Han Chinese cohort for further investigation and exploration. Second, this study was conducted exclusively on Han Chinese individuals, and the data obtained, particularly the genetic data, may not be applicable to other ethnic groups. Significant variations in the GC haplotypes have been observed across different populations, which could influence the measured genetic susceptibility risk related to vitamin D genetics (52–55). Third, environmental factors such as physical activity and sunlight exposure level that can influence serum 25(OH)D levels were not measured, which may affect the interpretations of the risk to vaccine-related myocarditis in this study. Furthermore, the vitamin D status of the included participants before vaccination would represent an important measurement for predicting the risk of this side effect. Nevertheless, measuring serum 25(OH)D level during the rapid-onset cardiac inflammatory period may still represent the baseline vitamin D status of patients prior to vaccination, as circulating 25(OH)D levels have a half-life of 15 days, hence, environmental factors affecting vitamin D level before and soon after the COVID-19 vaccination should be minimal (56).

In conclusion, our study provides the first evidence supporting the immunomodulatory role of vitamin D in reducing the risk and influencing the inflammatory cytokine response associated with unfavorable NK cell activation in BNT162b2 vaccine-related myocarditis. Our findings emphasize the importance of maintaining sufficient vitamin D levels, especially in those genetically susceptible to hypovitaminosis D, before receiving mRNA-based vaccines, which is likely to be the dominant vaccine platform in the future. Overall, the novel protective role of vitamin D in vaccine-related myocarditis uncovered in this study may pave the way for a new paradigm in understanding the biology of this unique side effect and devising new prevention and intervention strategies.

## Data Availability

The original contributions presented in the study are included in the article/**Supplementary Material**. Further inquiries can be directed to the corresponding author.
