# Supplementary material for: The protective role of vitamin D in BNT162b2 vaccine-related acute myocarditis
Source: Front Immunol. 2025 Feb 19;16:1501609. doi: 10.3389/fimmu.2025.1501609 (PMC11880265; doi:10.3389/fimmu.2025.1501609)
Supplement: Supplementary file 1 [file DataSheet1.docx]

**Supplementary Table 1. Characteristics of vaccine-related myocarditis convalescent patient samples**

|  | Convalescent Samples (N=9) |
| --- | --- |
| Median age in years | 16.0 |
| Age range | 12-17 |
| Gender (%)/ Median Age |  |
| Male | 7 (78.8%)/16.0 |
| Female | 2 (21.2%)/14.0 |
|  |  |
| Median Days after Diagnosis (Range) | 36.0 (16-59) |
| Collection Period | Sept 2021 - Dec 2021 |


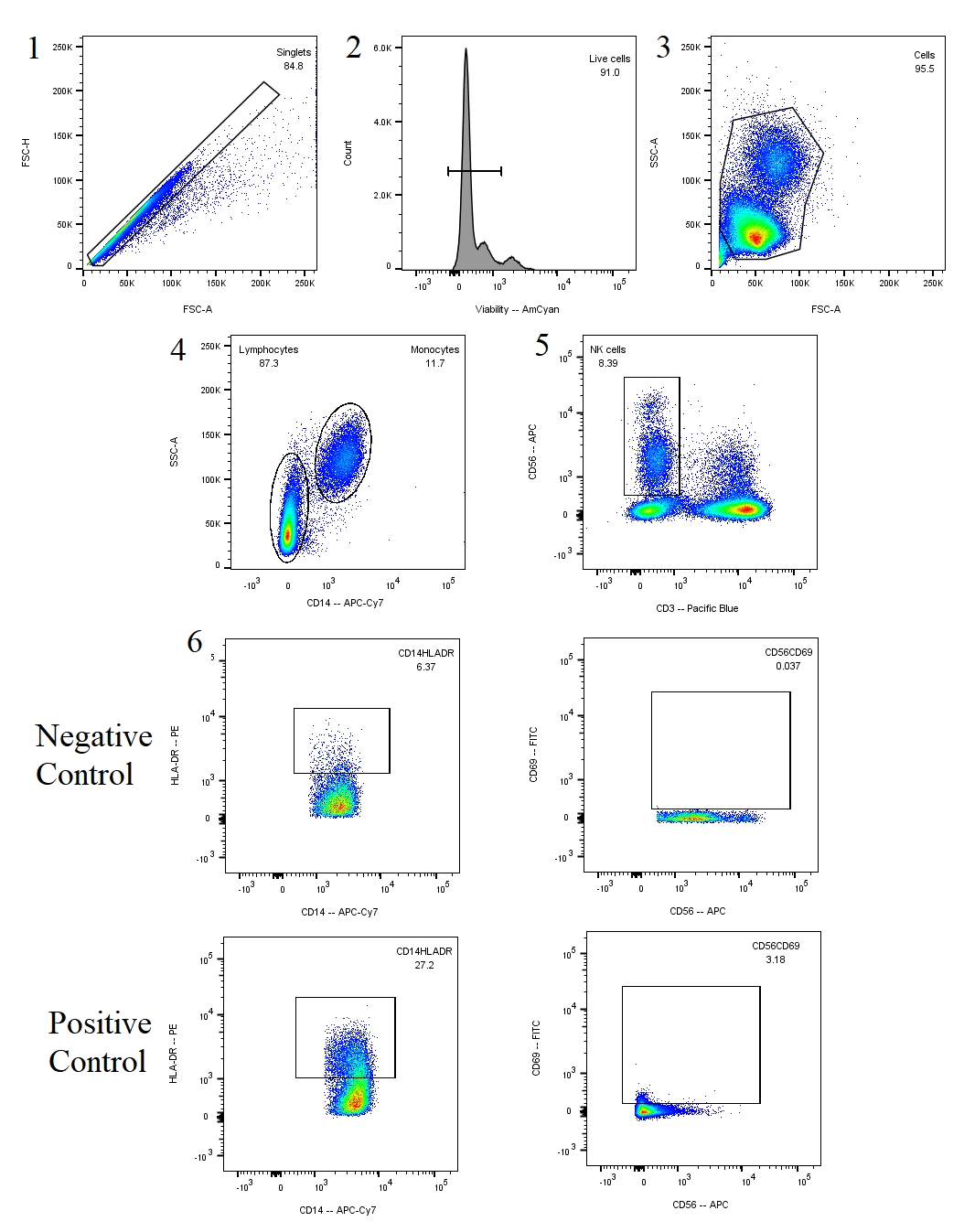


**Supplementary Figure 1. Representative gating analysis by flow cytometry. 1**. Forward scatter height (FSC-H) versus forward scatter area (FSC-A) plot for single cell inclusion. **2**. Live cells were gated based on live/dead discrimination dye staining. **3**. Side scatter area (SSC-A) versus forward scatter area (FSC-A) plot for lymphocyte and monocyte identification. **4**. Monocytes were gated based on specific high CD14 expression on the SSC-A versus CD14 plot, excluding lymphocyte populations. **5**. NK cells were gated from the specific high expression of CD56 from the gated lymphocyte population. **6**. Floating gate on HLA-DR, and CD69 on corresponding CD14 Monocytes; CD56 NK cells were identified and quantified based on the corresponding expression gating on positive control cells.


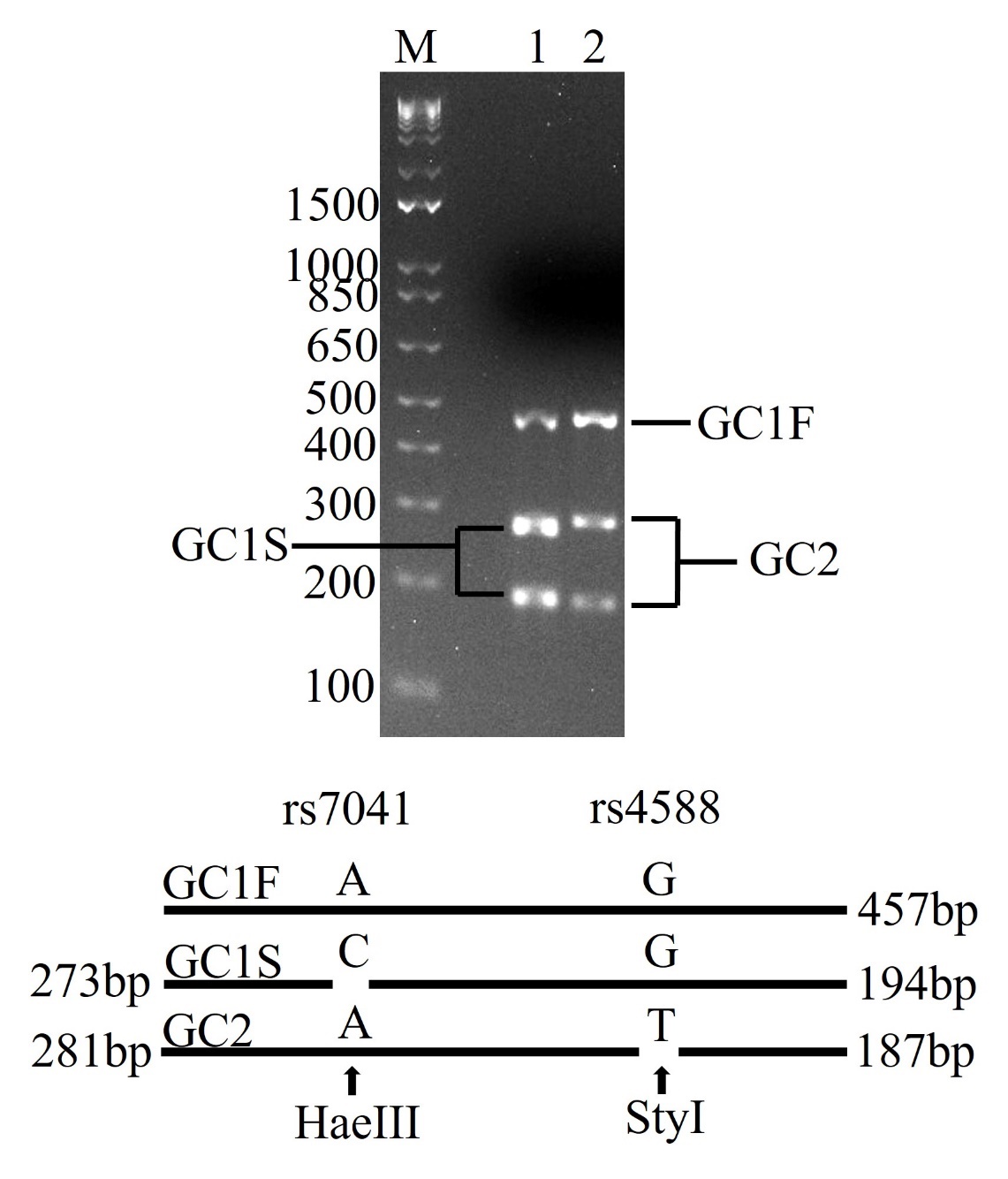

**Supplementary Figure 2. Representative diagram of GC isoform identification by Restrictive Fragment Length Polymorphism (RFLP).** HaeIII and StyI double-digested PCR products in heterozygous GC1F/S (Lane 1) and heterozygous GC1F/2 (Lane 2) carriers. GC isoforms indicated by the corresponding bands after 2% gel electrophoresis.
